# Clinical characteristics and hearing impairment in carriers of the m.3243 A > G variant

**DOI:** 10.1038/s10038-025-01412-3

**Published:** 2025-10-23

**Authors:** Natsumi Uehara, Takeshi Fujita, Keiichiro Uehara, Hikari Shimoda, Daisuke Yamashita, Jun Yokoi, Sayaka Katsunuma, Akinobu Kakigi, Ken-ichi Nibu

**Affiliations:** 1 Department of Otolaryngology-Head and Neck Surgery, Kobe University Graduate School of Medicine, Kobe, Japan; 2 Department of Diagnostic Pathology, Kobe University Graduate School of Medicine, Kobe, Japan

**Keywords:** Risk factors, Medical research

## Abstract

The m.3243 A > G mitochondrial DNA variant is a major pathogenic variant associated with various clinical phenotypes, including hearing impairment and diabetes. This study retrospectively analyzed clinical data from 37 patients with the m.3243 A > G variant to clarify the relationship between clinical characteristics and hearing loss. Most patients developed post-lingual, late-onset sensorineural hearing loss (SNHL), with flat-type audiometric configurations being the most common. Blood heteroplasmy levels were negatively correlated with age at genetic testing (R² = 0.6303), and age-adjusted heteroplasmy levels were inversely associated with age at onset of hearing loss (*p* = 0.029). A significant difference in clinical characteristics was observed between patients with hearing loss and/or diabetes alone and those with multiple organ involvement, with the latter group showing a lower BMI (*p* = 0.031) and more severe hearing loss (*p* < 0.001). Two cases initially presenting with maternally inherited deafness and diabetes progressed to MELAS (mitochondrial encephalopathy, lactic acidosis, and stroke-like episodes), demonstrating the importance of early systemic evaluation. Cochlear implantation was considered for advanced hearing loss, although systemic complications were a challenge. Our findings suggest that hearing loss may be an early risk factor of systemic mitochondrial disease, particularly in lean individuals. Comprehensive assessment, including BMI and genetic testing, may aid in the early diagnosis and management of patients with m.3243 A > G variant.

## Introduction

Mitochondrial diseases present with diverse symptoms, affect various organ systems, and show a wide range in age of onset and modes of inheritance. One key factor in this heterogeneity is mitochondrial heteroplasmy, a condition in which wild-type and mutant mitochondrial DNA (mtDNA) coexist in a single cell. The proportion of mutated mtDNA can range from 1% to 99% and can vary across different tissues, contributing to the broad phenotypic spectrum of mitochondrial diseases.

Clinical syndromes of mitochondrial disease include mitochondrial encephalopathy, lactic acidosis, and stroke-like episodes (MELAS); maternally inherited deafness and diabetes (MIDD); and chronic progressive external ophthalmoplegia. Other frequently reported features include isolated myopathy, cardiomyopathy, seizures, migraine, ataxia, cognitive impairment, gastrointestinal motility disorders, short stature, and hearing loss. Among these, sensorineural hearing loss (SNHL) is a prominent feature, reportedly affecting 30-90% of patients [1, 2]. In our previous genetic investigation of 48 cases of late-onset SNHL, the m.3243 A > G variant, recognized as one of the most prevalent pathogenic variants in mitochondrial disorders, was identified in 6 of the 48 cases (approximately 13%), making it the most frequently observed variant [3]. Hence, hearing loss is not only a principal symptom of mitochondrial disorders but also a notable cause of SNHL in general.

In this study, we focused on the clinical features in the m.3243 A > G carriers and examined their relationship with hearing impairment.

## Materials & Methods

This study was approved by the Ethics Committee of Kobe University Graduate School of Medicine (Approval No. 170081). Written informed consent was obtained from all participants. All procedures were performed in accordance with the Guidelines for Genetic Tests and Diagnoses in Medical Practice of the Japanese Association of Medical Sciences and the tenets of the Declaration of Helsinki.

### Subjects

Thirty-seven patients from 23 unrelated families who carry the m.3243 A > G variant, all of whom underwent medical examinations between April 2012 and April 2023 at Kobe University Graduate School of Medicine, participated in this study (Table 1).

**Table 1:** Clinical features of the m.3243 A > G carriers

**Number of subjects**	37
**Sex (n)**	
Female	17 (46%)
male	20 (54%)
**Heteroplasmy:% (range)**	<1–72
**Age adjusted Heteroplasmy (range)**	5–138
**Height:cm 20 y.o. and over (range:md)**	
Female	140–163.4 (150)
male	154.2–168.3 (163)
**BMI:kg/m**^**2**^ **(range:md)**	11.7–26 (18.3)
**Complications (n)**	
HL	32 (86%)
Diabetes	26 (70%)
heart disease	9 (24%)
kidney disease	6 (16%)
Developmental delays	1 (2.7%)
MELAS	6 (16%)
muscle weakness	6 (16%)
eye disease	2 (5.4%)
CPEO	1 (2.7%)
**Family history of HL(-) (n)**	3
**Age at genetic test:y.o. (range:md)**	5–78 (43)
**Age at onset of HL(n** = **32)**	
0–5 y.o.	0
6–10 y.o.	0
11–20 y.o.	11 (34%)
>20 y.o.	21 (66%)
**Age of using hearing aid:y.o. (range:md)**	13–67 (40)
**Severity of HL※ (n)**	
mild (21–40 dB)	11 (30%)
moderate (41–70 dB)	14 (38%)
severe (71–90 dB)	6 (16%)
profound (91dB-)	1 (2.7%)
normal (<20 dB)	5 (13%)
**Audiometric configuration (n)**	
Flat	16 (50%)
gently sloping	11 (35%)
steepy sloping	0
profound	1 (3%)
reverse U-shaped	4 (12%)

*HL* hearing loss, *y.o.* years old, *dB* decibel, *md* mean data,

*MELAS* mitochondrial myopathy, encephalopathy, lactic acidosis, and stroke-like episodes

*CPEO* chronic progressive external ophthalmoplegia

※Hearing level of worse side

### Clinical evaluations

A retrospective review was conducted of the following data: age at genetic testing, family history, heteroplasmy rate, age of onset of hearing loss (Age_HL), type of hearing device (if any), degree, type, and configuration of hearing loss, co-occurring symptoms, and body mass index (BMI). These clinical data were collected from medical charts.

Because the proportion of heteroplasmy in blood decreases with age, analyses examining its association with disease progression may be confounded by age. Therefore, in this study, we also evaluated the age-adjusted heteroplasmy rate [4, 5].

The age-adjusted heteroplasmy rate was calculated as described by Grady et al. [5], using the formula:

Age-adjusted heteroplasmy = Blood heteroplasmy / 0.977 ^(age+12)^

Hearing thresholds were evaluated using pure-tone audiometry (PTA), defined as the average of hearing thresholds measured at 500, 1000, 2000, and 4000 Hz. The severity of hearing loss was classified into four categories based on PTA: mild (21–40 dB HL), moderate (41–70 dB HL), severe (71–95 dB HL), and profound (>95 dB HL). The audiometric configurations were categorized as low-frequency type, reverse U-shaped, high-frequency type (gently sloping or steeply sloping type), flat type, or deaf, following a previous report [6].

### Statistical analysis

All statistical analyses and data visualization were performed using R software (version 4.4.2). We conducted a multivariate analysis of variance (MANOVA) using the ‘car‘ package, and generated visualizations using the ‘ggplot2‘ package. Multiple regression analyses were employed to examine the relationship between age-adjusted heteroplasmy and several independent variables, including Age_HL, BMI, sex, and hearing loss severity. The model was fitted using ordinary least squares regression, and *p*-values were corrected for multiple comparisons using the false discovery rate method. Observations with missing data were excluded. Statistical significance was set at *p* < 0.05.

## Results

### Patients characteristics

A total of 37 patients (21 male and 16 female) carrying the m.3243 A > G variant were studied (Table 1). The median age at genetic testing was 43 years (range: 5–78). Three patients (8%) had no family history of hearing loss. Among adult participants, females had heights ranging from 140.0 to 163.4 cm (median 150 cm), and males ranged from 154.2 to 168.3 cm (median 163 cm). BMI ranged from 11.7 to 26 kg/m^2^ (median 18.3 kg/m^2^).

### Hearing impairment

The age of onset of hearing loss was after language acquisition in all patients. Eleven cases (30%) developed hearing loss in their second decade of life (i.e.,11–19 years of age), and 23 developed hearing loss later (20–60 years of age), accounting for the majority of cases, indicating that late-onset hearing loss was common. To minimize the potential influence of presbycusis, we excluded two patients whose hearing loss began after age 60 from relevant analyses. Regarding audiogram configurations, flat-type SNHL was most common (16 cases), followed by gently sloping-type SNHL (11 cases). The degree of hearing loss varied widely (Table 1). On average, six cases had hearing thresholds below 20 dB HL and reported no subjective symptoms of hearing loss. However, they showed elevated thresholds at low-frequency tones and at 8 kHz. These findings suggest that these five cases may represent preclinical SNHL. In the statistical analysis, average hearing thresholds were also used for these cases.

**Fig. 1:**
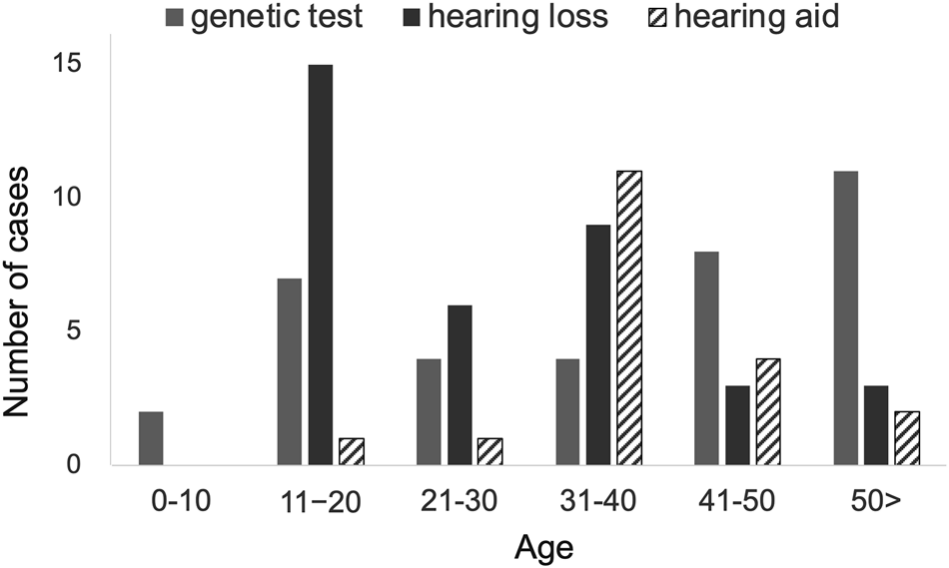
Age of onset of hearing loss. All cases showed hearing loss onset after language acquisition. Most patients began wearing hearing aids in their 30 s or later

In most cases, hearing loss developed after the age of 20, indicating that late-onset hearing loss was common. The average duration from the onset of hearing loss to the use of hearing aids was 12.2 years. Many patients started using hearing aids in their 30 s and 40 s (Fig. 1). Two patients were evaluated for cochlear implant (CI) surgery: one received a CI at the age of 79. The CI was effective, but speech perception gradually declined. He died of heart failure at the age of 82. A 62-year-old woman had severe heart failure, so she decided not to undergo CI surgery.

### Heteroplasmy level

**Fig. 2:**
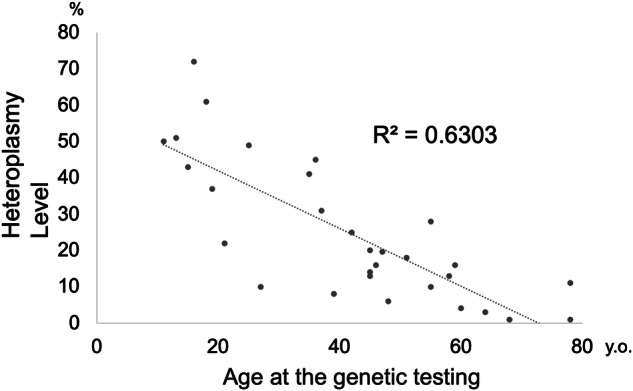
Heteroplasmy levels in blood. Heteroplasmy levels in lymphocytes decreased with increasing age at the genetic testing

Heteroplasmy levels of the m.3243 A > G variant were quantified in blood, which varied widely, ranging from below 1% to 72%. Three participants with no heteroplasmy data were excluded. A negative correlation was observed between variant load in blood and age at genetic testing (R^2^ = 0.6303) (Fig. 2), indicating that higher heteroplasmy levels were found in younger individuals.

Therefore, we also considered the age-adjusted heteroplasmy, ranging from 5 to 138%. We investigated potential factors influencing age-adjusted heteroplasmy via multiple regression analyses. Among the independent variables, Age_HL showed a significant negative association with this measure (*p* = 0.029, 95% CI [ − 0.266, −0.0154]), indicating that an increase in Age_HL was associated with a decrease in age-adjusted heteroplasmy. BMI exhibited a marginally significant negative association (*p* = 0.082). However, sex (*p* = 0.412), and hearing loss severity (*p* = 0.470) did not show statistically significant associations (Table 2).

**Table 2:** Factors Associated with age-adjusted heteroplasmy

Variable	Estimate (β)	Standard Error	95% CI (Lower, Upper)	*p*-value
Age_HL	−1.30	0.561	(−2.46, −0.14)	0.029
BMI	−4.72	2.60	(−10.1, 0.64)	0.082
sex	12.6	15.1	(−18.5, 43.7)	0.412
HL	0.248	0.338	(−0.450, 0.946)	0.470

*Age_HL* age of onset of hearing loss,

*BMI* body mass index,

*HL* hearing loss severity

### Clinical Features in Patients with the m.3243 A > G Variant

**Fig. 3:**
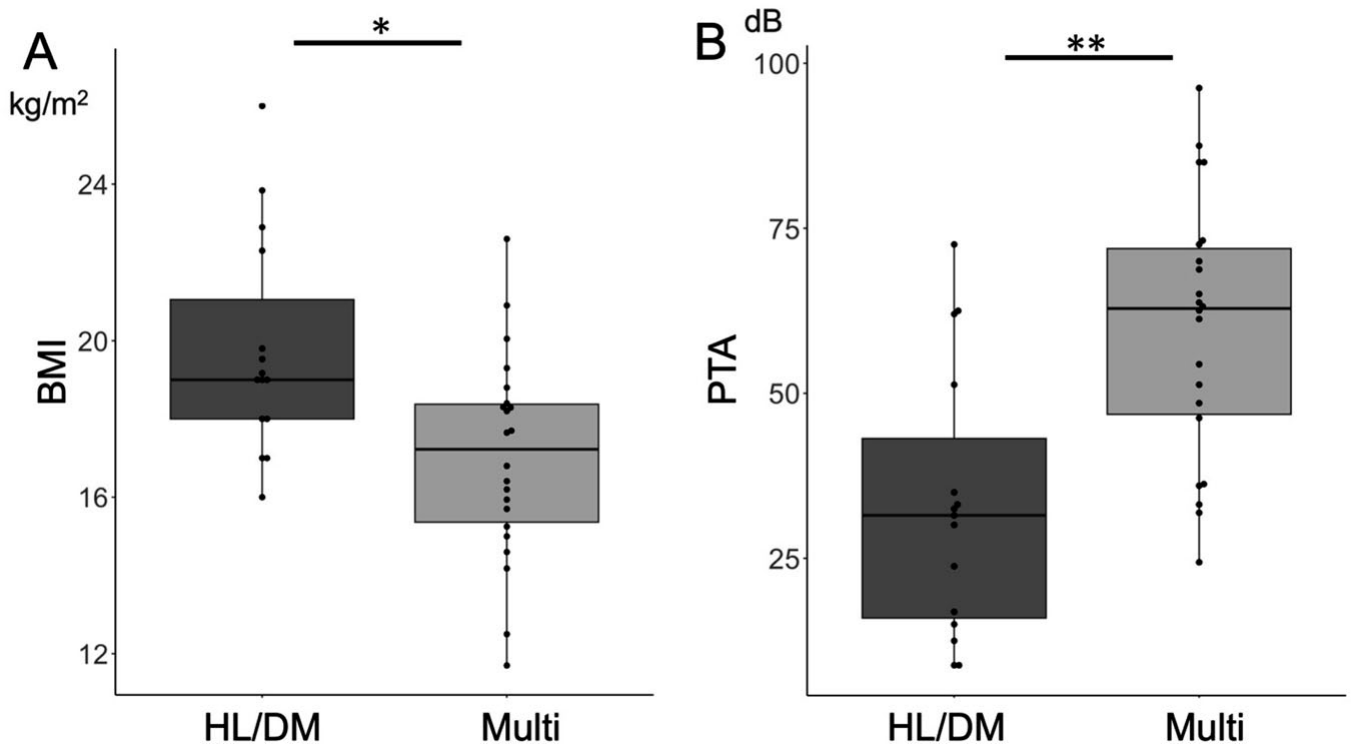
Characteristics of patients with multiple organ involvement. **A** Patients with multiple organ complications (Multi) had lower BMI than those with hearing loss and/or diabetes (HL/DM). **B** Multi group had more severe hearing loss than HL/DM group. *p < 0.05, **p < 0.001. BMI Body Mass Index, PTA pure-tone audiometry

Hearing loss was the most frequently observed symptom, followed by diabetes mellitus (Table 1). Of the 26 participants with diabetes mellitus, only 6 had MIDD, involving hearing loss and diabetes mellitus only. In 16 adult cases, symptoms were not limited to diabetes and hearing loss, but also involved multiple organ systems. This finding suggests that MIDD can progress to multi-organ complications. Therefore, we divided the cohort into two groups: the HL/DM group (*n* = 15), including only diabetes and/or hearing loss, and the Multiple group (*n* = 22), including patients with at least one organ system involvement in addition to hearing loss and diabetes. A Type II MANOVA was performed to evaluate the effect of organ involvement on multiple clinical variables, including BMI, Age_HL, heteroplasmy, age-adjusted heteroplasmy, and hearing loss severity. The MANOVA revealed a statistically significant multivariate effect of group (Wilks’ λ = 0.510, F (5, 23) = 4.42, *p* = 0.0058), indicating an overall difference in clinical profiles. Follow-up univariate analyses showed significant group differences in BMI (Fig. 3A: F = 5.20, *p* = 0.031) and hearing loss severity (Fig. 3B: F = 15.14, *p* < 0.001). A borderline difference was observed for heteroplasmy (*p* = 0.057), while no significant differences were detected for Age_HL or age-adjusted heteroplasmy (Supplementary 1).

### The progression cases from MIDD or hearing loss only to MELAS

Case 1 is a 55-year-old male (height 168.3 cm, BMI 18.8 kg/m^2^, the age-adjusted heteroplasmy rate 66%). He had no birth or developmental problems. He became aware of hearing loss in his 30 s, and began wearing bilateral hearing aids at the age of 50. He was diagnosed with diabetes at age 40 after experiencing thirst, polydipsia, and polyuria in his 30 s. He developed sudden right-sided hearing loss at age 55 after repeated falls and disorientation. MELAS was suspected because of his hearing loss, history of diabetes, and repeated cerebral infarctions, and genetic analysis identified m.3243 A > G, leading to the diagnosis of MELAS.

Case 2 is a 44-year-old female (height 150.0 cm, BMI 17.7 kg/m^2^, the age-adjusted heteroplasmy rate 26%). She became aware of hearing loss in her 30 s, and it gradually worsened. At around age 40, she experienced repeated episodes of headache. At 44, the patient presented with right hemiplegia, loss of speech, and aphasia, and a head MRI showed stroke-like findings. Given her mother’s history of diabetes and severe hearing loss, MELAS was suspected. Genetic testing revealed the m.3243 A > G variant. Subsequent systemic evaluation identified cardiomyopathy.

## Discussion

In this study, we analyzed 37 patients from 23 unrelated families carrying the m.3243 A > G variant. We aimed to identify the various complications that can arise in m.3243 A > G carriers, examine their heteroplasmy levels, characterize their clinical features, and explore their association with hearing impairment.

First, we examined the characteristics of hearing loss caused by m.3243 A > G variant. Hearing loss due to mitochondrial DNA variants can be classified into non-syndromic and syndromic hearing loss. The m.1555 A > G variant in the 12S rRNA gene is a common cause of non-syndromic hearing loss and is strongly associated with increased sensitivity to aminoglycoside antibiotics [7]. Audiometric patterns in patients with the m.1555 A > G variant typically show bilateral, sensorineural, symmetric, and high-frequency hearing loss. Hearing loss can also occur either with or without aminoglycoside exposure, and it can sometimes be progressive [8]. Some patients who have never received aminoglycosides still develop sudden sensorineural hearing loss, although it is often mild [9]. By contrast, the 3243 A > G variant in the tRNALeu (UUR) gene, commonly implicated in symptomatic hearing loss, can cause progressive, multi-organ damage, as seen in MIDD (diabetes and hearing loss) and MELAS (more severe, multi-system symptoms) [10]. Clinical findings that raise the suspicion of mitochondrial disease include maternal inheritance, deafness, diabetes, adult onset, short stature, and the involvement of multiple organ systems [11, 12]. Hearing loss can often be the first symptom in 30% to 40% of patients [13, 14]. In this study, the onset of hearing loss occurred after language acquisition in all patients, with 63.8% manifesting in adulthood (Table 1). A previous study reported that the median age of onset of hearing loss for m.3243 A > G is 25 years [11]. Few detailed investigations have examined the specific audiometric configuration [13, 15]. Liu et al. reported that among 44 MELAS patients, high-frequency hearing loss was the most common pattern (48%), followed by the flat-type (27%) and the low-frequency type (6%). Kuller et al. similarly reported mild-to-moderate high-frequency hearing loss (*n* = 5/11). In this study, however, flat-type hearing loss was the most frequent (16 of 32 cases, 50%), followed by gently sloping type (11 of 32, 34%), though patterns varied from one individual to another (Table 1). Previous reports may have focused on younger populations (median age in the 20 s), whereas our cohort had a higher median age (40 s). Progressive hearing loss over time may have led to flat-type configurations, even in cases that initially had high-frequency loss. Thus, in cases of SNHL, it is difficult to suspect that the hearing loss is due to mitochondrial disease based on the audiometric configuration type alone.

In MIDD, hearing loss often begins gradually, affects both ears, and may become severe over time [16]. Most patients start using hearing aids in their 30 s or later, around the same time they develop diabetes mellitus [2]. When hearing aids are no longer effective, cochlear implants (CI) are considered. Although some reports show that CI can be effective [17, 18], the risk of general anesthesia may be higher for patients with systemic conditions, such as those described in this study. Given that systemic deterioration may eventually limit surgical eligibility, early auditory intervention—ideally before the onset of major comorbidities—should be considered. Moreover, improvements in speech perception with CI may be limited or may gradually worsen due to retrocochlear involvement or higher brain dysfunction as neurological symptoms progress [18]. Therefore, timely referral for CI evaluation is essential, and before proceeding with CI, it is important to carefully assess the patient’s disease course and life expectancy.

Predicting the clinical course of m.3243 A > G-related disease is extremely difficult because the variant can produce a wide variety of symptoms. It typically shows a progressive trajectory and may initially be diagnosed solely as hearing loss or diabetes, which may not capture the full picture. Many studies have discussed factors associated with mitochondrial disease severity, including heteroplasmy. Indeed, several clinical investigations have examined the relationship between heteroplasmy and phenotype [1, 5, 19]. Disease phenotype has been correlated with age-adjusted heteroplasmy levels in blood, heteroplasmy levels, or urinary heteroplasmy levels [19]. However, the relationships between m.3243 A > G heteroplasmy levels and clinical features are often complex, since disease severity and progression can vary significantly from one individual to another. In this study, age-adjusted heteroplasmy tended to decrease as the age of onset of hearing loss increased. Although further studies with larger cohorts are warranted, it remains important to note that carriers with high age-adjusted heteroplasmy may develop hearing loss at an earlier age, even if they have not yet experienced it. These findings suggest that age-adjusted heteroplasmy may serve as a useful indicator of overall disease burden, especially in individuals with early-onset hearing loss. Continued follow-up in such cases could be clinically meaningful, even in the absence of other symptoms.

Additionally, we found that patients with multiple organ complications frequently have low BMI and progressive hearing loss. “Thin” cases need careful follow-up, as they may develop other symptoms in addition to hearing loss. Among m.3243 A > G-related disorders, MELAS is considered one of the most severe manifestations. Until recently, MELAS was managed primarily with symptomatic treatments, but L-arginine therapy was introduced to improve vascular endothelial function, and intravenous administration of L-arginine during both the acute and intermittent phases of stroke-like episodes has shown benefits for multiple stroke-related symptoms [20]. High-dose taurine therapy has also been shown to help prevent stroke-like episodes [21]. In our study, two patients who initially presented with hearing loss only or MIDD went on to develop MELAS seizures. Although neither patient was initially diagnosed with MELAS, early clinical suspicion enabled initiation of L-arginine therapy, which led to some improvement. Notably, both patients had a BMI close to 18, indicating a ‘thin’ body type. Although the age-adjusted heteroplasmy level was elevated in Case 1, it was not as high in Case 2. In this study, the association between BMI and age-adjusted heteroplasmy was marginally significant (*p* = 0.082). As age-adjusted heteroplasmy has been reported to correlate with disease progression, and we observed multiple organ complications—typically indicative of more severe disease—in patients with lower BMI, it is plausible that a significant association may emerge in larger cohort studies. We believe that this trend warrants further investigation.　This finding is consistent with our study results, which showed that a lower BMI is associated with multiple organ complications. Thus, although mitochondrial disease was once considered untreatable, recent breakthroughs in therapy and prevention hold promise. Therefore, recognizing the m.3243 A > G variant early can improve outcomes, particularly for patients with low BMI and progressively worsening hearing loss.

## Conclusion

When only hearing loss is noted at a certain point in time, it is difficult to recognize it as a potential mitochondrial disease. Consequently, identifying features such as low BMI, could contribute to an earlier diagnosis. To our knowledge, this is the first report describing the relationship between hearing loss in m.3243 A > G carriers and BMI.

Our findings also suggest that short stature and a thin body habitus may be a part of the clinical profile of mitochondrial disease. A comprehensive systemic examination, especially in younger patients, may facilitate earlier diagnosis, prevention, and appropriate treatment of serious complications such as MELAS syndrome.

## Supplementary information

Supplementary 1
