# Supplementary material for: Clinical characteristics and hearing impairment in carriers of the m.3243 A > G variant
Source: J Hum Genet. 2025 Oct 23;71(3):145–9. doi: 10.1038/s10038-025-01412-3 (PMC12948675; doi:10.1038/s10038-025-01412-3)
Supplement: Supplementary file 1 — Supplementary 1 [file 10038_2025_1412_MOESM1_ESM.pdf]

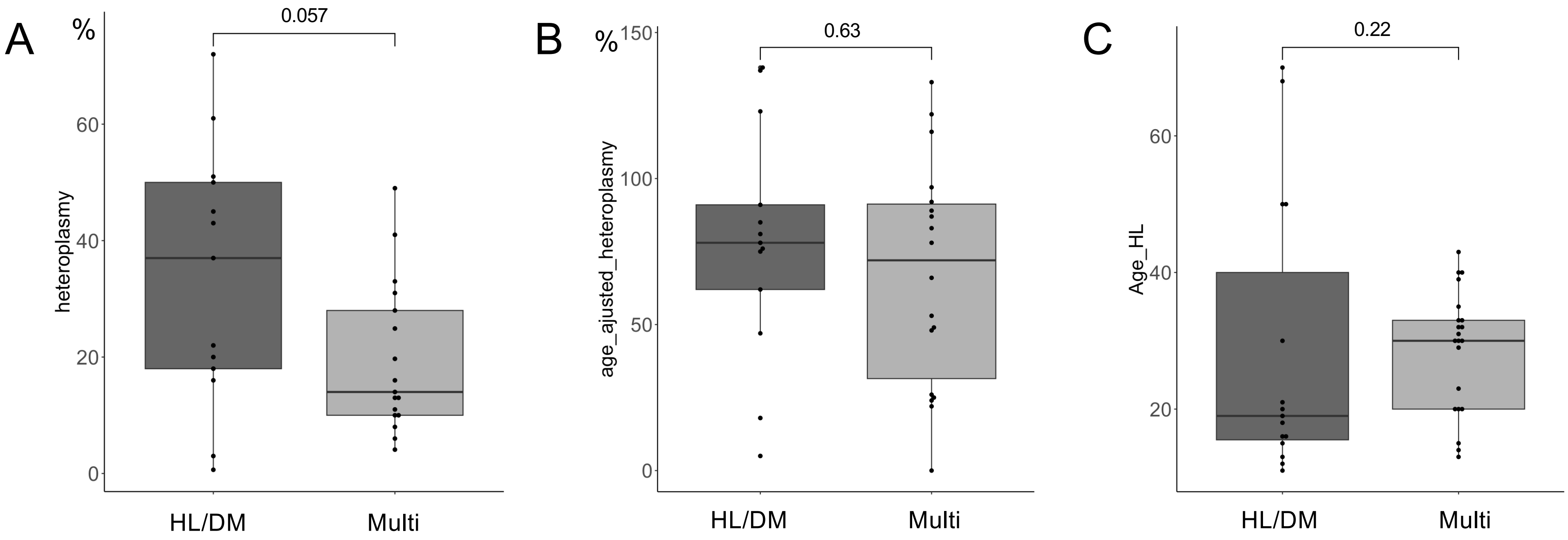

Supplementary1. **Comparison of Heteroplasmy, Age-Adjusted Heteroplasmy, Age at Hearing Loss Onset Between HL/DM and Multiple Groups.**

(A) A borderline difference was observed for heteroplasmy (p = 0.057).

(B) No significant difference was detected forage-adjusted heteroplasmy (p = 0.63).

(C) No significant difference was detected for age at hearing loss onset (p = 0.22).

Multi:Patients with multiple organ complications HL/DM: Patients with hearing loss and/or diabetes

Age_HL: age at hearing loss onset
